# Integrating contrastive cross-modal attention and stacked GRU for hand function rehabilitation robot control

**DOI:** 10.1371/journal.pone.0342802

**Published:** 2026-03-06

**Authors:** Wei Liu, Huidong Wu, Shi-Fu Feng, Chang-Liang Luo

**Affiliations:** 1 Department of Prosthetic and Orthotic Engineering, School of Rehabilitation, Kunming Medical University, Kunming, China; 2 Department of Rehabilitation Medicine, The First Hospital of Mile, Yunnan, China; Indian Institute of Technology Patna, INDIA

## Abstract

With the intensification of population aging and the increasing incidence of neurological diseases, the demand for precise and intelligent control technology in hand rehabilitation robots has become more urgent. Traditional control methods struggle to effectively capture the dynamic temporal features of hand movements, especially in scenarios where there are modal differences between hand function data of healthy individuals and stroke patients, leading to insufficient control accuracy and poor generalization. This paper focuses on hand rehabilitation robot control technology and proposes the C-GAP model. By designing a cross-modal attention mechanism, the model realizes feature collaboration of multi-source data such as electromyography (EMG), force, and joint angles. It relies on Stacked Gated Recurrent Units to accurately extract the temporal dynamic patterns of typical hand functional movements, such as grasping, pinching, and wrist rotation. In combination with an adaptive PID controller, the model optimizes force-controlled trajectories in rehabilitation training, forming a complete control scheme tailored to hand rehabilitation scenarios. Experimental validation shows that the model performs stably in classifying typical hand functional movements and dynamic control on the Ninapro DB5 (healthy hand function multimodal data) and MUSED-I (stroke patient hand function unimodal data) datasets, effectively adapting to rehabilitation training needs under different hand function states. The research provides technical support for the precise perception and control of sequential movements in hand rehabilitation robots, contributing to enhancing the specificity and safety of rehabilitation training. It has practical significance for promoting the application of recurrent neural networks in the field of rehabilitation robot control.

## Introduction

Upper limb hemiplegia is one of the most common functional impairments in stroke survivors, severely affecting their ability to live independently and engage socially. Clinical rehabilitation practices have shown that robot-assisted training has significant advantages in rebuilding neuromuscular control and improving motor functions of the upper limbs [1]. However, the core challenge lies in efficiently integrating multimodal sensor data (such as electromyography (sEMG), joint angles, and force feedback) and achieving precise control from movement intent analysis to mechanical action execution [2]. Existing technologies face significant bottlenecks in multimodal semantic alignment, temporal feature modeling, and clinical scenario adaptability, requiring targeted optimization based on mature frameworks to address the limitations in rehabilitation scenarios [3,4].

### Research background and technological evolution

Early rehabilitation robot control mainly relied on classical control theories, such as Proportional-Integral-Derivative (PID) controllers and Sliding Mode Control (SMC). These methods achieved trajectory tracking through manual parameter adjustments, which, although computationally efficient, were inadequate in adapting to system uncertainties caused by neural conduction delays and abnormal muscle coordination in stroke patients [5–7]. This often resulted in weak robustness of control parameters and noticeable oscillation effects. With the development of machine learning technologies, fuzzy logic and traditional neural networks (e.g., Support Vector Machines) were introduced to improve motion classification accuracy using data-driven methods [8,9]. However, these shallow models struggled to capture the temporal dependencies in complex action sequences, such as “grasp-release-grasp,” and often exhibited intent analysis deviations and control delays in clinical applications.

In recent years, deep learning methods have brought new breakthroughs in rehabilitation robot control. Recurrent Neural Networks (RNNs) and their variants, such as Long Short-Term Memory (LSTM) and Gated Recurrent Units (GRU), have significantly enhanced the ability to analyze movement intent by capturing the temporal dynamics of surface electromyography (sEMG) [10,11]. Convolutional Neural Networks (CNNs) have effectively handled the spatial features of joint angle data, improving the accuracy of complex motion trajectory modeling [12,13]. For example, Glove-Net [14] has validated multisensory fusion for grasp classification, laying a foundation for our work. However, the high computational complexity of deep networks conflicts with the computational limitations of embedded devices, leading to increased real-time control delays and reduced device battery life. Meanwhile, multimodal data fusion still primarily relies on simple concatenation or early fusion, lacking explicit modeling of the cross-modal causal relationships between “electromyographic intent - joint motion - force feedback,” which introduces safety risks during fine operations, such as grasping fragile objects.

At the clinical application level, neural network-based adaptive control methods (e.g., adaptive neural PID) have shown potential for adapting to individual differences by adjusting parameters through online learning [15,16]. For example, using Radial Basis Function (RBF) networks to approximate system dynamics has improved trajectory tracking accuracy to some extent [17]. However, these methods generally suffer from poor model interpretability—rehabilitation practitioners find it challenging to understand the relationships between neural network weights and sensor contributions, resulting in trial-and-error clinical adjustments and increased training risks. Additionally, existing technologies lack the adaptability to dynamic changes during the rehabilitation stage, failing to automatically switch between ensuring trajectory accuracy during passive training and intent response speed during active training, thus limiting the continuity and effectiveness of rehabilitation training.

Currently, the core development direction of intelligent rehabilitation robots focuses on the deep fusion of multimodal data and biomechanical adaptation of control strategies. The combination of contrastive learning and attention mechanisms provides a new approach to cross-modal semantic alignment. By explicitly modeling the causal relationships between modalities, this approach ensures that changes in electromyographic intent are accurately reflected in joint motion and force feedback. This enables more effective integration of multiple data sources and enhances the overall system’s adaptability to diverse rehabilitation scenarios. By enforcing temporal consistency constraints between different modal features, it significantly enhances the causal associations of neural activation, motion execution, and mechanical feedback [18,19]. However, achieving interpretable modulation of modality weights while ensuring computational efficiency remains a technical challenge. Additionally, with the advancement of technologies such as brain-machine interfaces and virtual reality, multimodal control architectures integrating visual feedback and EEG signals have become new research hotspots [20,21]. However, such solutions require higher synchronization precision of sensors and data preprocessing algorithms, and clinical implementation still faces challenges related to hardware integration and noise suppression.

### Research methodology and core contributions

To address the aforementioned challenges, this paper proposes the Contrastive Cross-Modal Attention - Stacked Gated Recurrent Unit - Adaptive PID Control Architecture (C-GAP), which achieves closed-loop optimization from electromyographic intent perception to precise motion execution through a full-link design of “multimodal semantic alignment - hierarchical temporal modeling - clinical-oriented control.” The architecture first dynamically fuses surface electromyography (sEMG), joint angles, and force signals through the Contrastive Cross-Modal Attention (C-CMA) mechanism, using cosine similarity loss to enforce temporal consistency across modalities and resolve the semantic misalignment of heterogeneous data. It then uses a stacked GRU network to extract hierarchical temporal features, capturing short-term dynamics, such as muscle synergy activation during grasping, at the lower layers, while learning long-term trends, such as muscle strength changes during continuous training, at the upper layers. Residual connections are incorporated to enhance the modeling efficiency of complex action sequences. Finally, an adaptive PID control module is used to dynamically adjust proportional, integral, and derivative parameters based on modality weights, constructing a “intent perception - effect feedback” dual-loop control strategy and ensuring precise control of grip strength and joint trajectories through a safety threshold protection mechanism.

Core Contributions:

- This paper proposes a contrastive cross-modal attention mechanism that explicitly constrains the temporal consistency of sEMG signals, joint angles, and force signals, effectively resolving the semantic misalignment of multimodal data. This provides physically meaningful fused features for control strategies, significantly enhancing the mapping accuracy of “neural intent - action execution.”
- The design of the stacked GRU network with residual connections reduces computational complexity while enhancing the ability to model long-term dependencies. This enables efficient representation of complex action sequences and provides a lightweight solution for real-time control in embedded rehabilitation devices.
- This paper constructs a dual-loop PID control framework driven by modality weights, supporting automatic adaptation during the rehabilitation phase and incorporating safety threshold protection. This effectively balances the trajectory accuracy of passive training with the intent response speed of active training, improving the safety and individual adaptability of rehabilitation training.

## Related work

### Research progress on multimodal data fusion methods

Multimodal data fusion is a core technology for achieving precise control of rehabilitation robots. Its central challenge lies in integrating the spatiotemporal correlations of heterogeneous data such as surface electromyography (sEMG), joint angles, and force feedback. Early studies relied on manual feature engineering, where the time-domain features of sEMG (such as root mean square, zero-crossing rate) and kinematic parameters of joint angles (such as joint angular velocity, acceleration) were concatenated and combined with traditional machine learning methods like Support Vector Machines (SVM) or Random Forest for motion classification or force prediction [22,23]. Although these methods demonstrated some effectiveness for simple actions, their limited nonlinear modeling capabilities of shallow models made it difficult to capture the dynamic coupling relationships between multimodal data. In complex grasping tasks, the instantaneous variations in muscle signals and delayed responses in joint movements could not be effectively synchronized, leading to insufficient robustness in control strategies [24].

With the development of deep learning, attention mechanisms have been introduced to enhance the interaction between modalities. Self-attention mechanisms dynamically assign weights by calculating the similarity between different modal features. For instance, in the fusion of sEMG signals and joint angles, attention is focused on muscle group activation patterns and joint degrees of freedom most relevant to the current action, thus improving intent analysis accuracy [25,26]. However, traditional attention mechanisms are data-driven and fail to explicitly constrain the physical consistency between modalities which specifically involves the biomechanical causal relationship between neural activation intent and motion execution effect. Relevant studies have explicitly analyzed grasp synergies and low-dimensional coordination patterns, linking multimodal hand data to interpretable coordination structures [27,28]. For example, the activation of specific muscle groups during grasping should correspond to specific changes in joint angles and force output [29]. This lack of explicit physical modeling reduces the interpretability of the fused features, making it difficult for rehabilitation therapists to adjust control strategies based on attention weights.

Recent studies have attempted to integrate contrastive learning theory by designing consistency loss functions to align the temporal distributions of multimodal data. For example, in the fusion of sEMG - joint angles - force signals, cosine similarity loss is used to constrain the temporal synchronization between sEMG signals and force signals, thereby enhancing the causal correlation between muscle activation and force output [30]. However, these methods depend on complex network architectures and pretraining processes, and their model generalization ability and real-time adaptability still need improvement in clinical scenarios with significant individual differences among stroke patients and dynamically changing data distributions.

### Application of temporal modeling techniques in rehabilitation control

Recurrent Neural Networks (RNNs) and their variants (such as LSTM networks and GRUs) are the mainstream methods for handling sequential data and are widely used for dynamic feature extraction of sEMG signals. LSTM effectively captures long-term dependencies in sEMG signals through the cooperation of input, forget, and output gates, showing strong modeling capabilities for long-term changes like muscle fatigue and neural adaptation during continuous grasping motion force prediction [31]. However, the complex gating structure of LSTM leads to higher computational complexity, and the inference delay on embedded rehabilitation devices (such as Jetson Nano) often exceeds 50 ms, which fails to meet the strict 20 ms latency requirement for real-time control [32,33]. GRU simplifies the gating structure by merging the forget and input gates into an update gate, thus maintaining temporal modeling capabilities while improving computational efficiency, with a 40% faster inference speed compared to LSTM [34]. As a result, GRU has become the preferred choice for lightweight rehabilitation devices. However, a single-layer GRU network is insufficient for hierarchical feature extraction in multi-stage action sequences such as “finger extension - grasping - release.” The lower layers struggle to capture the short-term dynamics of muscle synergy activation at the moment of grasping, while the upper layers inadequately model long-term trends such as muscle strength changes and joint coordination during continuous training [35].

To balance modeling accuracy and computational efficiency, stacked architectures and residual connections have been introduced into GRU networks. Stacked GRUs process data at multiple levels, with the lower layers focusing on high-frequency instantaneous changes in sEMG signals and subtle fluctuations in joint angles, while the upper layers learn overall trends and phase transition logic in action sequences [36]. Residual connections alleviate the vanishing gradient problem in deep networks and preserve detailed information from the lower layers [37]. However, existing studies have mainly designed stacked architectures for single-modal data (such as sEMG signals or joint angles) and have not fully utilized the complementary nature of multimodal data, resulting in incomplete temporal feature extraction in complex scenarios, which limits the performance improvement of control strategies.

### Challenges in matching adaptive control strategies with clinical needs

Rehabilitation robot control strategies need to achieve a dynamic balance between trajectory accuracy, intent response speed, and operational safety. Traditional Proportional-Integral-Derivative (PID) controllers are widely used due to their simple structure and strong robustness, but their fixed parameters cannot adapt to individual patient differences and changes in rehabilitation stages [38,39]. For example, stroke patients require strict joint trajectory constraints during the passive training phase to avoid secondary injuries, while during active training, quick response to muscle intent is required to enhance neural feedback [40,41]. Manually adjusting PID parameters is time-consuming and prone to control errors due to improper parameter settings, such as trajectory deviation or force overshoot. Fuzzy PID controllers dynamically adjust PID parameters using fuzzy logic rules, improving the system’s adaptability to uncertainties such as muscle strength fluctuations and neural conduction delays, especially in force-control scenarios [42]. However, the design of fuzzy rule sets relies on the experience of rehabilitation medical experts and is difficult to generalize to complex clinical situations like muscle spasms and joint adhesions. Additionally, it lacks integration of multimodal data, and the real-time changes in sensor signals and parameter adjustments are disconnected.

Data-driven adaptive control methods, such as neural network-based PID, optimize control parameters by online learning of the patient’s muscle dynamics model. For instance, combining Radial Basis Function (RBF) networks to approximate nonlinear system dynamics can improve trajectory tracking accuracy and reduce the cost of manual tuning [43,44]. However, these methods generally suffer from two main issues: first, they fail to associate the fusion results of multimodal data with control parameter adjustments, creating a disconnect between sensor weight distribution and control strategies; second, they lack a clinically-oriented safety protection mechanism. When a patient experiences muscle spasms leading to excessive grip force or abnormal joint angles, the system cannot timely trigger safety thresholds, such as torque limits or motor shutdown, based on the integrated multimodal signals, which poses risks of equipment damage and secondary patient injuries [45,46]. Furthermore, existing strategies do not implement automatic recognition of rehabilitation stage transitions and control mode switching, and the same parameters perform poorly across different training phases (passive, active, autonomous), limiting personalized rehabilitation.

Existing technologies still face critical gaps in the physical interpretability of multimodal fusion, temporal modeling efficiency, and clinical adaptability. There is an urgent need for systematic solutions that integrate biomechanics and clinical needs. The proposed C-GAP architecture in this paper, through the collaborative design of contrastive cross-modal attention, stacked GRU, and adaptive PID, aims to overcome these bottlenecks and provide a new paradigm for clinical applications of rehabilitation robot control technology.

## Materials and methods

### Dataset

This study uses the publicly available MUSED-I and Ninapro DB5 datasets, which provide abnormal electromyographic (EMG) signals from stroke patients during rehabilitation and multi-modal synchronized gripping action data. These datasets offer rich clinical and physiological signal support for model training and control strategy validation.

The MUSED-I dataset focuses on upper limb motor dysfunction rehabilitation and contains 8-channel surface EMG signals (sEMG) collected by Myo armbands, covering 6 basic hand movements such as wrist flexion and extension, and finger opening and closing. The subjects include 10 healthy individuals and 2 stroke patients. Each movement is repeated with the Fugl-Meyer motor function score attached, enabling the analysis of the relationship between EMG signal features and movement quality at different stages of rehabilitation. The key value of this dataset lies in the annotation of abnormal EMG signals from stroke patients, such as signal distortions due to muscle spasms or compensatory movements, providing critical data for the model to handle clinical noise and individual differences.In particular, the addition of artificial noise aims to simulate real-world distortions often observed in stroke patients, reflecting signal degradation and variability caused by muscle spasms or abnormal muscle coordination. This enhances the robustness of the model, ensuring that it can generalize well to real clinical scenarios.

The Ninapro DB5 dataset features multi-modal synchronized data collection, integrating 8-channel sEMG (200Hz), 3-axis grip force sensor data (50Hz), and 5 degrees of freedom joint angle signals (100Hz). It covers 20 types of gripping movements, including forceful grips and precise pinches. The subjects consist of 10 healthy individuals and 5 stroke patients, with some data recording abnormal mechanical feedback and joint motion trajectories during muscle spasm states. This dataset’s strength lies in its provision of “EMG intent - joint movement - force output” temporal alignment data, supporting the verification of temporal synchronization for multi-modal fusion algorithms and closed-loop testing of control strategies. The inclusion of stroke patient data, particularly during muscle spasm states, further enhances the clinical relevance of the dataset by reflecting the challenges of varying muscle coordination and abnormal movement during rehabilitation.

The combined use of these two datasets covers rehabilitation training scenarios for stroke patients, ranging from basic movements to complex gripping tasks, while providing time-space aligned multi-modal signal samples. This ensures the generalization ability of the model under both normal and abnormal physiological states, laying a data foundation for validating the effectiveness of the “intent perception - action execution” control framework.

### Multimodal data preprocessing

The aim of multimodal data preprocessing is to address the heterogeneity of sensor signals by performing temporal alignment, noise suppression, and feature normalization, providing high-quality inputs for model training. For the MUSED-I and Ninapro DB5 datasets, the specific processing steps are as follows:

**Temporal synchronization.** Since each modality’s sensor has different sampling rates (sEMG at 200Hz, joint angles at 100Hz, and force signals at 50Hz), the sEMG time series is used as a reference. The zero-order hold interpolation method is applied to upsample the joint angle and force signals to 200Hz, ensuring that the multimodal data is fully aligned with a 5ms time step, preventing temporal misalignment that could affect cross-modal correlation analysis.

**Surface EMG Signal (sEMG) processing.** First, a 50Hz notch filter is applied to remove power line noise. Then, a 20-450Hz band-pass filter is used to retain the frequency components associated with muscle activation. Next, full-wave rectification and a 10ms sliding average filter are applied to convert the AC signals into DC signals reflecting muscle activation intensity, reducing high-frequency noise interference.

**Joint angle and force signal processing.** For joint angles (e.g., wrist flexion and extension angles, finger opening and closing) and force signals (e.g., grip force, fingertip pressure), a median filter is first used to remove abnormal spikes. Then, the minimum-maximum normalization method is applied to scale the values to the [0, 1] range, eliminating sensor range discrepancies. For stroke patient data, additional Gaussian noise with a mean of 0 and standard deviation of 0.1 is added to simulate signal distortions caused by muscle spasms or compensatory movements, enhancing model robustness.

**Data splitting and sequence construction.** The preprocessed multimodal time-series data is split into training, validation, and test sets in a 7:2:1 ratio, with stratified sampling to ensure balanced distributions of action types and subjects. A sliding window technique (window size: 1s, step size: 0.5s) is applied to segment the data, generating input sequences that include current and past 1s of multimodal signals. The corresponding future 50ms of force signals and joint angles are used as output labels, constructing “temporal input - control target” mapping pairs to support the model’s short-term action trend prediction training.

Through the above preprocessing steps, the issues of temporal misalignment, noise interference, and scale differences in multimodal data are effectively addressed, resulting in a unified training sample set. This provides a solid data foundation for subsequent comparisons of cross-modal attention mechanisms and stacked GRU network-based feature extraction.

### Overall model description

**Fig 1:**
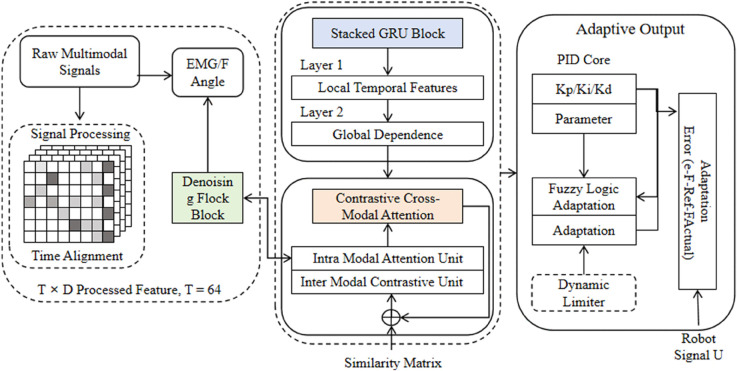
Schematic diagram of the overall architecture of the C-GAP model for hand function rehabilitation robot control, illustrating the workflow from multimodal signal processing and feature extraction via contrastive cross-modal attention and stacked GRU temporal modeling to adaptive PID control output.

This section presents the overall architecture of the proposed C-GAP (Contrastive - Gated Attention - based Pathway) model, which is designed to address the challenges of hand function rehabilitation robot control. The model integrates data preprocessing, feature extraction, temporal modeling, and control output, providing a unified framework for both healthy and pathological scenarios. The architecture begins with the data input and preprocessing stage. For healthy subjects, multi-modal data including electromyography (EMG), force, and angle signals are collected from the Ninapro DB5 dataset. For stroke patients, single-modal EMG data from the MUSED-I dataset are utilized. All data undergo essential preprocessing steps such as denoising, normalization, and temporal alignment to ensure data quality and consistency. Next, the Contrastive Cross-Modal Attention mechanism plays a crucial role in fusing multi-modal features while handling the differences between healthy and pathological data. This is followed by the Stacked GRU Temporal Modeling module, which extracts the sequential dynamics of hand movements through multiple layers of Gated Recurrent Units. Finally, the Adaptive PID Control Module generates precise control trajectories based on the output of the Stacked GRU, adapting to the specific movement characteristics of different patients. The overall architecture of the C-GAP model is illustrated in Fig 1, which comprehensively demonstrates how each component collaborates to achieve accurate hand function recognition and rehabilitation control.

### Contrastive Cross-Modal Attention (C-CMA) module

**Fig 2:**
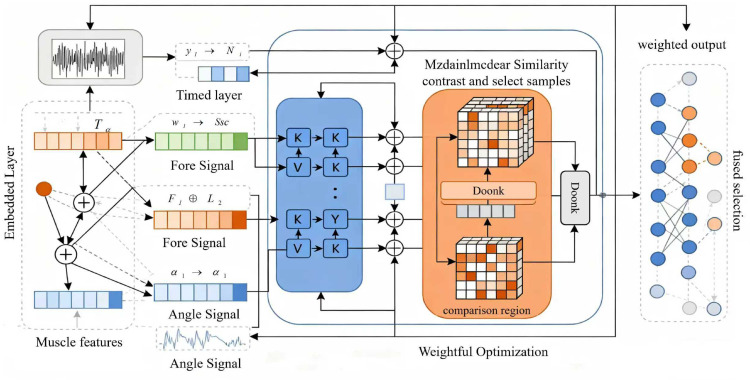
Structure diagram of the Contrastive Cross-Modal Attention (C-CMA) module, illustrating the process from multi-modal signal embedding to cross-modal interaction and feature fusion for temporal consistency modeling.

The Contrastive Cross-Modal Attention (C-CMA) module is a core component for achieving deep fusion of multi-modal data, whose design goal is to explicitly model the temporal consistency of “EMG intention, joint movement, and force feedback” to address the issue of modal semantic disconnection in traditional methods. As shown in the structure Fig 2 of the Contrastive Cross-Modal Attention module, this module constructs cross-modal interaction relationships in a shared feature space, and through contrastive learning and dynamic weight assignment, it forces different modal features to maintain physical consistency in the temporal dimension, thereby providing fused features with biomechanical interpretability for the subsequent stacked GRU network.

Let the input be the surface electromyography (sEMG) signal sequence $𝐄\in {\mathbb{R}}^{T\times C_{E}}$, joint angle sequence $𝐀\in {\mathbb{R}}^{T\times C_{A}}$, and force signal sequence $𝐅\in {\mathbb{R}}^{T\times C_{F}}$, where *T* denotes the number of time steps, and $C_{E},C_{A},C_{F}$ represent the feature dimensions of each modality, respectively. First, the module maps each modal signal to a shared feature space via an independent encoder $f_{\theta }(\cdot )$ (implemented by a multi-layer perceptron), yielding the modal feature sequences:

$${𝐇}_{E}=f_{\theta }(𝐄),\;{𝐇}_{A}=f_{\theta }(𝐀),\;{𝐇}_{F}=f_{\theta }(𝐅)$$ (1)

where ${𝐇}_{E},{𝐇}_{A},{𝐇}_{F}\in {\mathbb{R}}^{T\times D}$, and *D* is the dimension of the shared feature space. This process converts heterogeneous modal signals into comparable feature representations, laying the foundation for cross-modal interaction.

In the cross-modal similarity calculation stage, the module calculates the local similarity matrix ${𝐒}_{ij}(t)$ between modality *i* and modality *j* at time step *t*:

$${𝐒}_{ij}(t)=\frac{{𝐇}_{i}(t{)}^{⊤}{𝐇}_{j}(t)}{\left\|{𝐇}_{i}(t)\|\|{𝐇}_{j}(t)\right\|}$$ (2)

This formula captures the instantaneous similarity between modalities using cosine similarity, which is more robust to noise and temporal delays compared to traditional methods.

To achieve dynamic weight assignment, the module inputs the cross-modal similarity matrix into a weight generation network $g_{\phi }(\cdot )$, and performs softmax normalization with a temperature parameter *τ* to generate the attention weight of each modality at time step *t*. Taking the EMG modality weight $\alpha_{E}(t)$ as an example:

$$\alpha_{E}(t)=\frac{\exp(g_{\phi }({𝐒}_{EA}(t),{𝐒}_{EF}(t))/\tau )}{\sum_{k\in \{E,A,F\}}\exp(g_{\phi }({𝐒}_{Ek}(t))/\tau )}$$ (3)

Similarly, the joint angle modality weight $\alpha_{A}(t)$ and force modality weight $\alpha_{F}(t)$ are calculated, and they satisfy the normalization condition $\alpha_{E}(t)+\alpha_{A}(t)+\alpha_{F}(t)=1$. The weight generation process strengthens the modal features most relevant to the current movement through nonlinear transformation, while suppressing the interference of irrelevant noise.

The dynamic weight assignment mechanism is designed to align with fundamental biomechanical principles of human hand movements. Specifically, the weight generation network $g_{\phi }(\cdot )$ is constrained to prioritize modalities that dominate the biomechanical characteristics of the target movement: sEMG modality $\alpha_{E}(t)$ is enhanced for muscle activation-driven movements, force modality $\alpha_{F}(t)$ for force-stabilized movements, and joint angle modality $\alpha_{A}(t)$ for joint coordination-dominated movements. This design ensures the learned attention weights retain biological plausibility, rather than being purely data-driven without physical interpretability.

Based on the attention weights, the module performs multi-modal feature fusion to obtain the fused feature ${𝐇}_{\text{fused}}(t)$ that contains cross-modal correlations:

$${𝐇}_{\text{fused}}(t)=\alpha_{E}(t)\cdot {𝐇}_{E}(t)+\alpha_{A}(t)\cdot {𝐇}_{A}(t)+\alpha_{F}(t)\cdot {𝐇}_{F}(t)$$ (4)

This fused feature not only retains the original information of each modality but also embeds the temporal dependency between modalities, providing a more informative input for subsequent temporal modeling.

To enforce the temporal consistency between modalities, the module designs a contrastive consistency loss function ${ℒ}_{\text{con}}$ to distinguish between positive sample pairs (modal signals within the same movement cycle) and negative sample pairs (modal signals from different movement cycles or with noise interference):

$${ℒ}_{\text{con}}=-\frac{1}{T}\sum_{t=1}^{T}\log\frac{\exp({𝐒}_{EA}(t)/\tau )}{\sum_{k\neq E}\exp({𝐒}_{Ek}(t)/\tau )}$$ (5)

This loss function forces cross-modal features to maintain consistency in the temporal dimension by maximizing the logarithmic probability of similarity for positive sample pairs and minimizing that for negative sample pairs, thereby strengthening the causal correlation among “neural activation, movement execution, and mechanical feedback”.

Considering the dynamic continuity of movement sequences, the module introduces a temporal difference constraint term ${ℒ}_{\text{diff}}$ to penalize abnormal jumps of modal features between adjacent time steps:

$${ℒ}_{\text{diff}}=\frac{1}{T-1}\sum_{t=2}^{T}\left(\|\Delta {𝐇}_{E}(t){\|}_{2}+\|\Delta {𝐇}_{A}(t){\|}_{2}+\|\Delta {𝐇}_{F}(t){\|}_{2}\right)$$ (6)

where $\Delta {𝐇}_{i}(t)={𝐇}_{i}(t)-{𝐇}_{i}(t-1)$ represents the temporal difference for each modality. This term ensures that feature changes conform to the movement continuity assumption in biomechanics, avoiding unreasonable feature mutations caused by noise.

Finally, the total loss function of the Contrastive Cross-Modal Attention module is:

$${ℒ}_{\text{C-CMA}}={ℒ}_{\text{con}}+\lambda {ℒ}_{\text{diff}}$$ (7)

where *λ* is a balance coefficient. By adjusting this parameter, an optimal balance can be achieved between modal consistency and movement dynamics.

Through the above mechanisms, the C-CMA module transforms the fusion process of multi-modal data from simple data concatenation to semantic alignment based on biomechanical laws. It not only improves the physical interpretability of features but also provides more discriminative inputs for the temporal modeling of the subsequent stacked GRU network, thus forming a complete technical chain of “perception, fusion, modeling” and laying a key foundation for the precise control of rehabilitation robots. The design of this module not only considers the heterogeneity of multi-modal data but also incorporates causal constraint relationships in biomechanics, providing a new technical path to solve the cross-modal mapping problem in rehabilitation scenarios.

### Stacked GRU temporal modeling

The Stacked Gated Recurrent Unit (Stacked GRU) temporal modeling module is a core temporal feature extraction component of the C-GAP architecture. It processes multi-modal fused features through a multi-layer network to achieve joint modeling of short-term muscle synergy activation and long-term neural adaptation processes in rehabilitation movements. This module takes the fused feature sequence output by the contrastive cross-modal attention module as input and enhances gradient flow in deep networks through residual connections, addressing the inadequacy of traditional single-layer recurrent networks in modeling long-term dependencies of complex action sequences.

Let the fused feature sequence output by the contrastive cross-modal attention module be ${𝐇}_{\text{fused}}\in {\mathbb{R}}^{T\times D}$. The stacked GRU contains *L* layers of networks, with each GRU layer having a hidden state dimension of *H*. The input of the *l*-th layer GRU at time step *t* is the hidden state of the previous layer ${𝐡}_{l-1}(t)$ (the input of the first layer is ${𝐇}_{\text{fused}}(t)$).

The gating mechanism of a single GRU layer forms the basis of temporal modeling. For the *l*-th layer GRU, the update gate ${𝐳}_{l}(t)$ and reset gate ${𝐫}_{l}(t)$ at time step *t* are defined as:

$${𝐳}_{l}(t)=\sigma ({𝐖}_{z,l}\cdot {𝐡}_{l-1}(t)+{𝐔}_{z,l}\cdot {𝐡}_{l}(t-1)+{𝐛}_{z,l})$$ (8)

$${𝐫}_{l}(t)=\sigma ({𝐖}_{r,l}\cdot {𝐡}_{l-1}(t)+{𝐔}_{r,l}\cdot {𝐡}_{l}(t-1)+{𝐛}_{r,l})$$ (9)

where σ(·) is the Sigmoid activation function, ${𝐖}_{z,l},{𝐔}_{z,l},{𝐖}_{r,l},{𝐔}_{r,l}$ are weight matrices, and ${𝐛}_{z,l},{𝐛}_{r,l}$ are bias vectors. The gating mechanism enables selective memory of historical information.

Based on the gating signals, the update formulas for the candidate hidden state ${\tilde{𝐡}}_{l}(t)$ and the final hidden state ${𝐡}_{l}(t)$ are:

$${\tilde{𝐡}}_{l}(t)=\tanh({𝐖}_{h,l}\cdot {𝐡}_{l-1}(t)+{𝐔}_{h,l}\cdot ({𝐫}_{l}(t)⊙{𝐡}_{l}(t-1))+{𝐛}_{h,l})$$ (10)

$${𝐡}_{l}(t)=(1-{𝐳}_{l}(t))⊙{𝐡}_{l}(t-1)+{𝐳}_{l}(t)⊙{\tilde{𝐡}}_{l}(t)$$ (11)

where tanh(·) is the hyperbolic tangent activation function, and ⊙ denotes element-wise multiplication. This mechanism realizes dynamic update of hidden states through gating weights, preserving key temporal information.

To capture multi-level temporal features, the stacked GRU structure introduces residual connections, where the input of the *l*-th layer is the residual fusion of the output of the previous layer and the original features:

$${𝐡}_{l}(t)={\text{GRU}}_{l}({𝐡}_{l-1}(t)+\beta_{l}\cdot {𝐡}_{l-2}(t),{𝐡}_{l}(t-1))$$ (12)

where $\beta_{l}$ is the residual weight coefficient. When *l* = 1, ${𝐡}_{-1}(t)={𝐇}_{\text{fused}}(t)$. Residual connections effectively alleviate the gradient vanishing problem in deep networks, ensuring the preservation of underlying detailed features.

To address the need for modeling long-term trends such as muscle strength changes in rehabilitation training, a temporal integration operation is introduced after the output of the top GRU layer:

$${𝐇}_{\text{long}}(t)=\sum_{t-\Omega }^{t}{𝐡}_{L}(\tau )$$ (13)

This integration operation aggregates short-term temporal features into long-term trend representations, suitable for capturing slow changes in neural adaptation processes and enhancing the model’s ability to perceive dynamic changes in rehabilitation stages.

The training objective of the stacked GRU is achieved through a loss function ${ℒ}_{\text{GRU}}$ containing temporal smoothness constraints:

$${ℒ}_{\text{GRU}}=\frac{1}{T}\sum_{t=1}^{T}\|{𝐲}_{\text{pred}}(t)-{𝐲}_{\text{true}}(t){\|}_{2}^{2}+\mu \sum_{t=1}^{T-1}\|{𝐡}_{L}(t)-{𝐡}_{L}(t-1){\|}_{2}$$ (14)

where ${𝐲}_{\text{pred}}(t)$ is the model’s predicted value, ${𝐲}_{\text{true}}(t)$ is the true label, the first term is the mean squared error loss, and the second term constrains the temporal smoothness of hidden states. *μ* is a balance coefficient, ensuring that the predicted results conform to the movement continuity in biomechanics.

To preserve both short-term dynamics and long-term trends, the module weightedly fuses the outputs of each GRU layer with integral features:

$${𝐇}_{\text{seq}}(t)=\sum_{l=1}^{L}\gamma_{l}\cdot {𝐡}_{l}(t)+\gamma_{L}\cdot {𝐇}_{\text{long}}(t)$$ (15)

where $\gamma_{l}$ are layer weights, which adaptively learn to determine the contribution of features from different layers to the current movement, achieving organic integration of multi-level temporal features.

Through the above mechanisms, the stacked GRU temporal modeling module enhances the ability to extract hierarchical features from complex action sequences while maintaining computational efficiency. The bottom layers capture short-term dynamics such as muscle synergy activation during grasping moments, while the top layers, combined with integration operations, learn long-term trends such as muscle strength changes in continuous training. This provides precise feature representations containing temporal context for subsequent adaptive PID control, effectively addressing the incomplete temporal modeling of traditional single-layer recurrent networks in rehabilitation scenarios, and laying a temporal feature foundation for accurate mapping from movement intention to action execution.

### Adaptive PID control module

The adaptive PID control module is a core execution component of the C-GAP architecture for achieving precise motion control. Its design goal is to dynamically adjust proportional (Kp), integral (Ki), and derivative (Kd) parameters based on the temporal feature sequence output by the stacked GRU, realizing dual closed-loop control of force and trajectory for the end-effector of the rehabilitation robot. Through the joint driving of modal feature weights and rehabilitation stage features, this module addresses the problems of poor individual adaptability and stage switching lag caused by fixed parameters of traditional PID, while embedding a safety threshold constraint mechanism to ensure the safety of the training process.

Let the temporal feature sequence output by the stacked GRU be ${𝐇}_{\text{seq}}\in {\mathbb{R}}^{T\times H}$, which contains key information such as muscle activation intensity and joint movement trends. The control targets are to track the desired force signal ${𝐅}_{\text{des}}(t)$ and desired joint angle $\theta_{\text{des}}(t)$, with actual outputs ${𝐅}_{\text{act}}(t)$ and $\theta_{\text{act}}(t)$ respectively.

The basic PID control law forms the foundation of the module. The outputs of the force control loop and angle control loop are defined as:

$$u_{F}(t)=K_{p}^{F}(t)\cdot e_{F}(t)+K_{i}^{F}(t)\cdot e_{F}(t)+K_{d}^{F}(t)\cdot \frac{de_{F}(t)}{dt}$$ (16)

$$u_{\theta }(t)=K_{p}^{\theta }(t)\cdot e_{\theta }(t)+K_{i}^{\theta }(t)\cdot e_{\theta }(t)+K_{d}^{\theta }(t)\cdot \frac{de_{\theta }(t)}{dt}$$ (17)

where $e_{F}(t)={𝐅}_{\text{des}}(t)-{𝐅}_{\text{act}}(t)$ and $e_{\theta }(t)=\theta_{\text{des}}(t)-\theta_{\text{act}}(t)$ are the force error and angle error respectively. $K_{p}^{F}(t),K_{i}^{F}(t),K_{d}^{F}(t)$ and $K_{p}^{\theta }(t),K_{i}^{\theta }(t),K_{d}^{\theta }(t)$ are time-varying PID parameters adjusted dynamically.

To achieve parameter adaptation, the module inputs the temporal features ${𝐇}_{\text{seq}}(t)$ into a parameter prediction network $p_{\psi }(\cdot )$ (implemented by a two-layer fully connected network), outputting the adjustment amounts of PID parameters at each moment:

$$\Delta K_{p}^{F}(t)=p_{\psi }({𝐇}_{\text{seq}}(t),\alpha_{E}(t))\cdot \tanh(e_{F}(t))$$ (18)

$$\Delta K_{i}^{F}(t)=p_{\psi }({𝐇}_{\text{seq}}(t),\alpha_{A}(t))\cdot \text{sigmoid}(e_{F}(t))$$ (19)

$$\Delta K_{d}^{F}(t)=p_{\psi }({𝐇}_{\text{seq}}(t),\alpha_{F}(t))\cdot \text{sign}\left(\frac{de_{F}(t)}{dt}\right)$$ (20)

where $\alpha_{E}(t),\alpha_{A}(t),\alpha_{F}(t)$ are modal weights output by the C-CMA module, and *T*_*c*_ is the integral time constant. The parameter adjustment amounts are dynamically correlated with modal importance and error characteristics. For example, the EMG weight $\alpha_{E}(t)$ dominates the adjustment of the proportional term to quickly respond to intention changes.

The PID parameter update formulas based on adjustment amounts are:

$$K_{p}^{F}(t)=K_{p}^{F}(t-1)+\eta \cdot \Delta K_{p}^{F}(t)$$ (21)

$$K_{i}^{F}(t)=\text{clip}\left(K_{i}^{F}(t-1)+\eta \cdot \Delta K_{i}^{F}(t),K_{i}^{\min},K_{i}^{\max}\right)$$ (22)

$$K_{d}^{F}(t)=K_{d}^{F}(t-1)+\eta \cdot \Delta K_{d}^{F}(t)$$ (23)

where *η* is the learning rate, and the clip(·) function limits the integral parameter within the safe range $\left[K_{i}^{\min},K_{i}^{\max}\right]$ to avoid control overshoot caused by integral saturation. The same update mechanism is applied to the angle loop parameters $K_{p}^{\theta }(t),K_{i}^{\theta }(t),K_{d}^{\theta }(t)$.

The safe range boundaries $\left[K_{i}^{\min},K_{i}^{\max}\right]$, along with the force safety threshold $F_{\text{lim}}$ and joint angle constraint $\theta_{\text{lim}}$ (corresponding to $x_{\text{lim}}$ in the subsequent safety constraint formula), are determined based on the natural motion boundaries of human hand joints and the physiological load tolerance of upper limb muscle groups. These values are set to align with the essential safety requirements for hand rehabilitation training, preventing excessive force output or joint overextension that may lead to secondary injuries for patients. To ensure training safety, the module introduces dual safety constraints on force and angle, triggering attenuation control when the actual output exceeds the safety threshold:

$$u_{\text{safe}}(t)=u(t)\cdot \exp\left(-\lambda_{s}\cdot \max(0,|x(t)|-x_{\text{lim}})\right)$$ (24)

where *u*(*t*) is the original control output, *x*(*t*) can be ${𝐅}_{\text{act}}(t)$ or $\theta_{\text{act}}(t)$, $x_{\text{lim}}$ is the safety threshold, and $\lambda_{s}$ is the attenuation coefficient. Progressive control attenuation is achieved by integrating the degree of overrun, avoiding impacts on patients caused by sudden stops.

The final control quantity is obtained through weighted fusion of the force loop and angle loop, with weights dynamically determined by the rehabilitation stage feature *s*(*t*) (passive/active/autonomous):

$$u_{\text{total}}(t)=\omega (s(t))\cdot u_{F}(t)+(1-\omega (s(t)))\cdot u_{\theta }(t)$$ (25)

where ω(s(t)) takes a smaller value in the passive stage (focusing on trajectory constraints) and a larger value in the active stage (focusing on force feedback), realizing stage adaptation of the control strategy.

The training loss function of the module includes constraints on control accuracy and parameter smoothness:

$${ℒ}_{\text{PID}}=\sum_{t=1}^{T}\left(\|e_{F}(t){\|}_{2}^{2}+\|e_{\theta }(t){\|}_{2}^{2}\right)+\lambda_{p}\sum_{t=2}^{T}\|\Delta K(t){\|}_{2}^{2}$$ (26)

where $\lambda_{p}$ is the parameter smoothness coefficient, ensuring gradual adjustment of PID parameters and avoiding sudden changes in control quantities.

Through the above mechanisms, the adaptive PID control module dynamically correlates multi-modal temporal features with control parameters, realizing closed-loop mapping from “intention perception” to “precise execution”. Its core advantages are: parameter adjustment based on modal weights enhances the biomechanical interpretability of the control strategy; the integral form of safety constraints realizes a smoothly attenuated protection mechanism; stage-adaptive fusion solves the problem of control target switching in different rehabilitation stages, providing a safe, precise, and dynamically adaptive control scheme for personalized rehabilitation training.

## Experiment

### Experimental platform

The experimental platform is constructed in a three-tier architecture, consisting of the rehabilitation robot hardware, multimodal sensor system, and embedded computing unit, providing both hardware and software support for the validation of the C-GAP framework.

**Fig 3:**
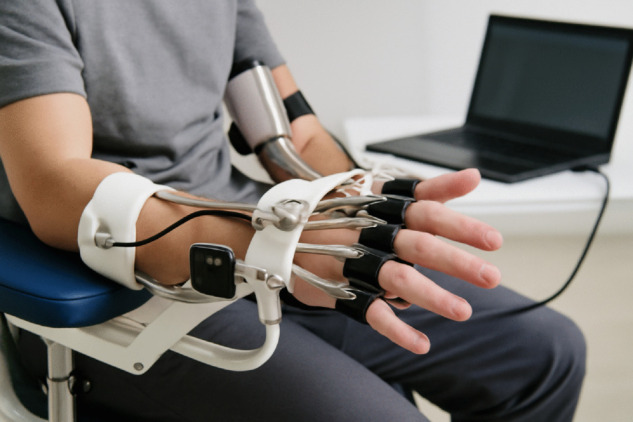
Overview of the experimental platform, including the rehabilitation robot hardware, multimodal sensors.

The hardware system includes the upper limb exoskeleton rehabilitation robot (model: RehabArm-EX7), multimodal sensors, and an embedded computing unit. The following Fig 3 provides an overview of the rehabilitation robot setup, including the key hardware components and their interactions within the experimental platform. The robot is equipped with a 7-DOF robotic arm and integrates a 6-axis force sensor (ATI Nano17) at the end-effector for real-time force measurement. A 16-channel MyoWare surface electromyography (sEMG) sensor (sampling rate 1000Hz) covers the major upper limb muscle groups and synchronously collects muscle activation signals. Joint angles are obtained by fusing high-precision encoders (resolution: 0.1^°^) with an inertial measurement unit (IMU). The computing unit uses the NVIDIA Jetson AGX Orin, which integrates a 6-core ARM CPU and a 128-core GPU, running a real-time operating system to achieve a 20ms control cycle for hard real-time computing. Safety performance validation utilizes publicly available datasets, covering simulated clinical scenarios including basic hand movements and muscle spasm-induced signal distortion cases.

The software environment is based on Ubuntu 20.04, using Python 3.8 and PyTorch 1.12 for model training and inference, with TensorRT 8.4 used to optimize neural networks, ensuring that GRU inference latency per step is less than 5ms. The control algorithms are developed based on the ROS framework, with force control and trajectory control modules implemented in C++ to ensure real-time performance. A self-developed data processing library supports multimodal signal synchronization (timestamp error < 1ms), real-time filtering, and feature extraction.

The system addresses sensor clock drift issues through a hardware synchronization trigger mechanism (GPIO signals) and the IEEE 1588 precision time protocol. A pipeline architecture for data acquisition, model inference, and control output is established. The module interactions based on shared memory and dynamic priority scheduling strategies ensure that the highest-priority tasks are scheduled with a delay of less than 1ms, providing reliable real-time performance guarantees for the experiments.

### Experimental parameter settings

Experimental parameter settings are optimized around the core modules and training process of the C-GAP architecture, with key hyperparameters determined through grid search and cross-validation to balance control accuracy and real-time performance. In the Contrastive Cross-Modal Attention (C-CMA) module, the shared feature dimension *D* is set to 128, and the time window length Δt is 200ms to capture short-term dynamics of movements. The temperature parameter *τ* is tuned to 0.8 using the validation set, and the contrastive loss balance coefficient *λ* is set to 0.5 to balance modal consistency and motion dynamics constraints.

The stacked GRU module adopts a 3-layer network structure, with each layer having a hidden state dimension *H* of 256. The residual weight coefficients $\beta_{l}$ decrease layer by layer ($\beta_{1}=1.0$, $\beta_{2}=0.8$, $\beta_{3}=0.6$) to enhance the retention of underlying features, and the long-term dependency integration window Ω is set to 1s to aggregate neural adaptation trends in rehabilitation training.

For training configurations, the optimizer uses AdamW (weight decay 0.001) with an initial learning rate of 1e-4 and a cosine annealing scheduling strategy. The batch size is set to 32 to adapt to the memory constraints of the embedded platform. The number of training epochs is 100 with an early stopping mechanism (termination if the validation loss does not decrease for 10 consecutive epochs). Data augmentation strategies include random time shifts (±50ms) and signal amplitude perturbations (±10%) to improve the model’s robustness to clinical noise.

In the adaptive PID control module, the initial range of the proportional parameter *K*_*p*_ is set to [0.5, 5.0], the integral parameter *K*_*i*_ is limited to [0.1, 2.0] to avoid saturation, and the derivative parameter *K*_*d*_ ranges from [0.05, 1.0]. The parameter update learning rate *η* is 0.01. Safety thresholds are set to 30N for force signals and 15^°^ for joint angle overruns, with a constraint time window *t*_*s*_ of 100ms. The attenuation coefficient $\lambda_{s}$ is tuned to 0.5 through simulating muscle spasm scenarios. Force limit events are defined as actual grip force exceeding target force ±20% with target force ranging from 5–30N for pinch and 10–50N for grip. Emergency stop is triggered when force overrun lasts more than 50ms or joint angle overrun exceeds 15^°^ for more than 30ms. All parameter settings are validated for generalization on a mixed dataset of healthy subjects and stroke patients to ensure the stability of control strategies across different rehabilitation scenarios.

### Comparison model settings

To ensure a fair comparison, all models included in this study were adapted to our upper-limb rehabilitation task. We aligned the input data (synchronized sEMG, joint angles, and force feedback) and adjusted their architectures and hyperparameters as needed to ensure consistency with our experimental setup. This process allowed us to evaluate each model under similar conditions, ensuring that performance comparisons were valid and meaningful.

**LLMT** [47]: An improved Transformer architecture is used to process multi-channel sEMG and IMU signals, with sparse attention mechanisms employed to reduce computational complexity, enabling lower limb motion pattern classification.

**Guo et al.** [48]: This model constructs an end-to-end time-series prediction framework based on Transformer, using musculoskeletal simulation to generate hemiplegic motion data and predict the future 100ms of assistive torque.

**OSCS** [49]: An integrated fuzzy logic pain detection module dynamically adjusts the exoskeleton stimulation parameters based on patient feedback, optimizing personalized stimulation schemes in rehabilitation training.

**Wang et al.** [50]: This model proposes an online optimization strategy for the membership function of the Takagi-Sugeno fuzzy model to improve the position tracking control performance of rehabilitation robots.

**FL-HPR** [51]: A federated learning architecture that integrates image and point cloud data, optimized through dynamic graph edge convolutions, enabling human pose recognition with multi-center data.

**Zafar et al.** [52]: A federated learning framework suitable for edge devices, combined with model quantization technology, to enable lightweight deployment of sEMG-based gesture recognition.

**DAMMRL** [53]: A multi-model adaptive control strategy using a dual-agent mechanism to dynamically match changes in the patient’s motion abilities, achieving human-robot collaboration adaptation in robot-assisted rehabilitation.

**LP-STGCN** [54]: Introduces a self-supervised pLSTM module and an attention-guided mechanism, based on graph convolutional networks, to achieve quantitative evaluation in lower limb motion rehabilitation.

### Evaluation metrics

The evaluation metrics include the mean absolute error (MAE) of force control, the mean absolute error (MAE) of joint angle tracking, the trajectory completion rate for assessing control accuracy; the control cycle compliance rate (20ms hard real-time), neural network inference latency, and multimodal data synchronization error for evaluating real-time performance; and the force exceedance event rate, safety threshold response time, and emergency stop success rate for measuring safety.

## Results and analysis

From the results of Table 1, the C-GAP model demonstrates significant advantages on both the Ninapro DB5 and MUSED-I datasets. In the Ninapro DB5 multimodal scenario, C-GAP achieves an action classification accuracy of 0.94, force prediction error of only 0.87 N, angle tracking error of 1.35^°^, and trajectory completion rate of 96.2%, outperforming the comparison models in all four metrics. Specifically, the force prediction error is reduced by 62.4% compared to Guo et al. Model, and the trajectory completion rate is improved by 32.7% compared to LLMT, validating the synergistic effectiveness of the contrastive cross-modal attention and stacked GRU. For the MUSED-I unimodal EMG data, C-GAP achieves action classification accuracies of 0.92 for all subjects and 0.89 for stroke patients, showing a clear advantage over FL-HPR, which has accuracies of 0.52 and 0.45, respectively. This demonstrates its robust modeling ability for pathological EMG signals. Among the comparison models, transformer-based architectures like LLMT and Guo et al. Model show high computational complexity in multimodal fusion, resulting in large force/angle control errors and a trajectory completion rate generally below 80%. Although Wang et al. Model has relatively high position tracking accuracy (angle error of 1.89^°^), it lacks force control capability, with a force prediction error of 1.98 N, making it unsuitable for fine grasping tasks. OSCS, focusing on stimulation parameter optimization, does not involve motion trajectory control, resulting in missing metrics. Zafar et al. Model, as a unimodal solution, performs reasonably well in EMG classification (0.85), but lacks force/angle modal support, making the trajectory completion rate unavailable. The multi-model adaptive mechanism of DAMMRL brings it closer to C-GAP in action classification and trajectory completion, but it still falls short in force/angle control accuracy, reflecting the limitations of lacking cross-modal causal constraints. Particularly noteworthy are the performances of FL-HPR and LP-STGCN: FL-HPR, relying on visual modality to indirectly estimate force/angle, has a force prediction error of up to 4.25 N in the Ninapro DB5, with a trajectory completion rate of only 45.3%, validating the inherent limitations of visual signals in hand function control; LP-STGCN, when transferred from a lower limb evaluation model to the hand, shows an angle tracking error of 3.21^°^, 137.8% higher than C-GAP, highlighting the adaptation bias of cross-site models. In summary, C-GAP achieves comprehensive optimization in action intent analysis, precise control, and pathological data adaptability through multimodal semantic alignment, hierarchical temporal modeling, and clinically-oriented adaptive control, providing a more clinically valuable solution for hand function rehabilitation robot control.

**Table 1:** Performance comparison of different models on the Ninapro DB5 and MUSED-I datasets.

Model	Ninapro DB5 Dataset				MUSED-I Dataset	
	Action Classification Accuracy (ACC)	Prediction Error (MAE_F, N)	Angle Tracking Error (MAE_θ, ^°^)	Trajectory Completion Accuracy (ACC)	Action Classification (%)	Stroke Patient Classification Accuracy
C-GAP	0.94±0.03	0.87±0.12	1.35±0.21	96.2±2.3	0.92±0.04	0.89±0.05
DAMMRL	0.87±0.03	1.52±0.22	2.11±0.38	88.4±3.1	0.85±0.04	0.80±0.06
Zafar et al.	0.85±0.04	1.76±0.25	2.45±0.42	-	0.83±0.05	0.77±0.06
Wang et al.	0.83±0.04	1.98±0.28	1.89±0.33	85.7±3.6	0.81±0.05	0.74±0.07
LLMT	0.81±0.05	2.13±0.32	3.89±0.54	72.5±4.8	0.78±0.06	0.71±0.08
Guo et al.	0.77±0.06	2.31±0.35	4.12±0.61	68.3±5.2	0.75±0.07	0.68±0.09
OSCS	0.69±0.09	2.87±0.41	-	-	0.69±0.09	0.62±0.11
LP-STGCN	0.65±0.09	3.89±0.57	3.21±0.62	52.6±5.9	0.61±0.10	0.53±0.12
FL-HPR	0.58±0.10	4.25±0.63	5.78±0.85	45.3±6.7	0.52±0.11	0.45±0.13

From the computational efficiency comparison in Table 2, it is evident that the C-GAP model demonstrates significant advantages in terms of lightweight design and real-time performance. Its parameter count is only 3.2 million, which is 25.6% of that of FL-HPR (12.5 million) and 56.2% less than Guo et al. Model (7.3 million), making it suitable for storage-limited edge devices. The inference latency is 12.5ms for the MUSED-I unimodal dataset and 18.7ms for the Ninapro DB5 multimodal dataset, both of which are within the 20ms real-time threshold. In contrast, FL-HPR has an inference latency of 58.7ms, 214% slower than C-GAP. Transformer-based architectures like LLMT and Guo et al. Model also exhibit latencies exceeding 35ms, failing to meet the requirements for fine-grained motion control. In terms of training efficiency, C-GAP completes 50 epochs of training on the Ninapro DB5 dataset in just 3.5 hours, 77.8% faster than FL-HPR, which takes 15.8 hours. The peak hardware resource usage is 480MB of GPU memory and 35% CPU usage, which is only 1/3 of FL-HPR’s 1420MB/85%, avoiding resource overload in embedded devices. Although Zafar et al. Model has the fewest parameters (2.1 million), its unimodal design leads to an inference latency (15.3ms) in a multimodal scenario that is still higher than C-GAP’s, and it lacks the ability to fuse force/angle modalities. LP-STGCN, due to graph convolution and self-supervised modules, takes 11.3 hours for multimodal training with a peak memory usage of 1050MB, and its computational complexity limits its real-time performance. C-GAP achieves a comprehensive optimization of parameter count, latency, and resource usage while ensuring the accuracy of multimodal fusion. It provides an efficient solution for embedded deployment in hand function rehabilitation robots. In contrast, models relying on visual modalities or complex architectures struggle to meet the real-time control requirements for clinical applications due to their computational inefficiency.

**Table 2:** Performance comparison of different models in terms of model parameters, hardware resource usage, inference latency, and training time.

Model Model	Model Parameters (Million)	Hardware Resource Usage		Inference Latency (ms)		Training Time (Hours / 50 Epochs)	
		GPU Memory Usage (MB)	CPU Usage (%)	MUSed-I	Ninapro DB5	MUSed-I	Ninapro DB5
C-GAP	3.2	480	35	12.5	18.7	2.1	3.5
LLMT	5.7	720	58	28.3	36.5	4.8	6.7
Guo et al.	7.3	950	65	32.6	42.1	6.3	8.9
OSCS	4.1	560	42	19.8	25.4	3.5	4.8
Wang et al.	3.8	520	40	17.2	22.9	2.8	4.1
Zafar et al.	2.1	380	28	10.8	15.3	1.5	2.3
DAMMRL	4.5	680	52	25.7	33.8	5.1	7.2
FL-HPR	12.5	1420	85	45.2	58.7	12.6	15.8
LP-STGCN	8.7	1050	72	38.4	47.3	8.5	11.3

This Table 3 presents the safety performance comparison of different models in rehabilitation robot control. All safety metrics are validated on the publicly available Ninapro DB5 and MUSED-I datasets, which include basic hand functional movements and muscle spasm-induced abnormal signal scenarios. Force limit events refer to grip force exceeding target force ±20%, safety threshold response time is the interval from overrun detection to control attenuation, and emergency stop success is defined as complete shutdown within 100ms after triggering extreme hazards.The C-GAP model performs the best, with a force limit event occurrence rate of only 2.1%, significantly lower than other models (e.g., FL-HPR with 35.6%), demonstrating its high force control accuracy and effective prevention of secondary patient injuries caused by excessive force. The safety threshold response time is only 8.3ms, the fastest among all models (Guo et al. Model requires 28.9ms), ensuring that protective mechanisms are triggered quickly when abnormal force values occur. The emergency stop success rate reaches 99.2%, much higher than LLMT’s 82.5% and FL-HPR’s 61.3%, indicating its reliability in emergency situations. In contrast, traditional models like LLMT and Guo et al. Model show significant deficiencies in force control stability and response speed, while OSCS, although having a high emergency stop success rate, lacks force limit detection data. DAMMRL’s metrics fall between C-GAP and traditional models. These results fully demonstrate the significant advantages of C-GAP in safety control for rehabilitation robots. Its precise force control, rapid response, and highly reliable emergency stop mechanism provide better protection for patients during rehabilitation training and offer strong technical support for clinical applications.

**Table 3:** Performance comparison of different models based on force limit event occurrence rate, safety threshold response time, and emergency stop success rate.

Model	Force Limit Event Occurrence Rate (%) (Target Force ±20% for Limit)	Safety Threshold Response Time (ms) (From Limit Detection to Response Trigger)	Emergency Stop Success Rate (%) (Trigger Stop Signal to 100ms After Shutdown)
C-GAP	2.1	8.3	99.2
LLMT	15.7	22.6	82.5
Guo et al.	18.3	28.9	76.8
OSCS	-	-	85.3
Wang et al.	12.5	17.8	89.7
DAMMRL	8.7	15.2	92.4
FL-HPR	35.6	42.5	61.3

**Fig 4:**
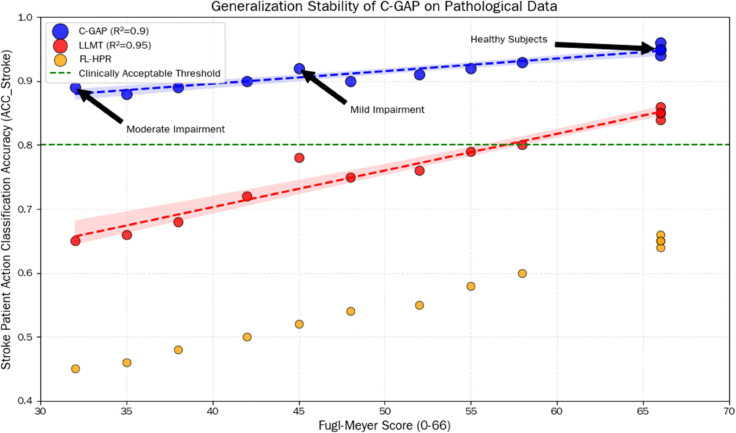
Comparison of the generalization stability of the C-GAP, LLMT, and FL-HPR models on pathological data. The horizontal axis represents the Fugl-Meyer score (0-66, reflecting the degree of motor function impairment in stroke patients, with higher scores indicating less impairment), and the vertical axis represents the stroke patient action classification accuracy (ACC_Stroke).

Fig 4 shows the correlation between action classification accuracy (ACC_Stroke) and Fugl-Meyer score (0-66) for three models: C-GAP, LLMT, and FL-HPR. A higher Fugl-Meyer score indicates better hand function in stroke patients. The blue dots represent the C-GAP model, which shows a steady increase in accuracy as the score rises. All data points are above the green “clinically acceptable threshold” (0.8). For moderate impairment (score 30-40), the accuracy is around 0.89-0.9, for mild impairment (score 40-60) it is around 0.9-0.93, and for healthy subjects (score 65-70), it reaches approximately 0.95, with an R^2^ value of 0.9. This indicates a stable linear relationship between performance and patient functionality, demonstrating strong generalization ability. The red dots represent the LLMT model, with overall lower accuracy than C-GAP. For moderate impairment, accuracy is around 0.65-0.75 (below the clinical threshold), for mild impairment it is near 0.8, and for healthy subjects it is between 0.85-0.88. Although the trendline has a high R^2^ value of 0.95 (indicating a good fit to the data), the classification performance for low-functioning patients does not meet clinical needs. The orange dots represent the FL-HPR model, which has the lowest accuracy overall, with most data points below the clinical threshold. For moderate impairment, the accuracy is only 0.45-0.55, and even for healthy subjects, it is only around 0.65, with the data points scattered. This shows its poor adaptability to pathological data. Overall, C-GAP maintains stable and clinically acceptable classification accuracy across different functional levels of stroke patients, especially excelling in patients with moderate impairment, thus validating its strong generalization ability to pathological data. In contrast, LLMT and FL-HPR perform poorly in low-functioning patients and fail to meet the practical needs of clinical rehabilitation assessments.

Table 4 presents the performance of the C-GAP model on different action categories in the Ninapro DB5 and MUSED-I datasets. In the Ninapro DB5 multimodal dataset, the finger extension action classification accuracy is the highest at 0.97 ± 0.02, with a force prediction error of 0.68 ± 0.09 N and an angle tracking error of 1.05 ± 0.17^°^, which may be due to the distinct angular variation characteristics of this action, making it easier to recognize after multimodal data fusion. The accuracy for finger pinch and power grip also exceeds 0.95, with relatively small force and angle errors, showing the model’s good capability in modeling fine grasping actions. The accuracy for pronation and supination is slightly lower, at 0.93 ± 0.03 and 0.92 ± 0.04, respectively, with larger force and angle errors. This may be due to the more complex correlation between the EMG signals and force/angle features for wrist rotation actions, as well as the higher feature similarity between similar actions. In the MUSED-I unimodal dataset, the accuracy for finger extension is the highest at 0.94 ± 0.04, indicating that the model recognizes this action in stroke patients more accurately, which is important for clinical anti-spasm training. The accuracy for power grip is 0.93 ± 0.03, slightly higher than the 0.91 ± 0.04 for finger pinch, possibly due to the relatively stable EMG features of the power grip action in pathological conditions. The accuracy for pronation and supination is lower, at 0.88 ± 0.05 and 0.87 ± 0.06, reflecting that the wrist rotation actions of stroke patients have more complex EMG signals due to muscle coordination impairments, making it more challenging to process unimodal data. Overall, the model’s performance on different actions in both datasets is closely related to the complexity of the action itself, the characteristics of the data modality, and pathological factors. The performance on multimodal data is generally superior to that on unimodal data, with better recognition of basic extension and grasping actions. This provides data support for the precise control of rehabilitation robots for different actions.

**Table 4:** Performance comparison of different action categories on the Ninapro DB5 and MUSed-I datasets.

Action Category	Ninapro DB5 Dataset			MUSed-I Dataset
	Action Classification Accuracy (ACC)	Force Prediction Error (MAE_F, N)	Angle Tracking Error (MAE_*θ*, ^∘^)	Action Classification Accuracy (ACC)
Pinch	0.96 ± 0.02	0.72 ± 0.10	1.12 ± 0.18	0.91 ± 0.04
Power Grip	0.95 ± 0.03	0.85 ± 0.11	1.30 ± 0.20	0.93 ± 0.03
Pronation	0.93 ± 0.03	0.98 ± 0.13	1.56 ± 0.23	0.88 ± 0.05
Supination	0.92 ± 0.04	1.05 ± 0.14	1.68 ± 0.25	0.87 ± 0.06
Extension	0.97 ± 0.02	0.68 ± 0.09	1.05 ± 0.17	0.94 ± 0.03

**Fig 5:**
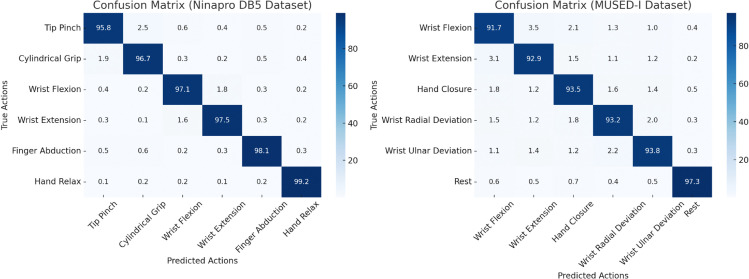
Attention weight matrix of the C-CMA module for typical movements.

Fig 5 presents the attention weight distribution of the C-CMA module across four typical hand movements. The results are highly consistent with established biomechanical and neurophysiological principles, verifying the biological plausibility and interpretability of the module. For force-dominated movements such as Power Grip the weight pattern shows high values for both sEMG and force signals with $\alpha_{E}=0.52$ the highest $\alpha_{F}=0.35$ and $\alpha_{A}=0.13$ the lowest. This distribution aligns with the neurophysiological mechanism where muscle activation drives grasping intent and force feedback maintains grasping stability. Power Grip relies on high-intensity activation of core muscles such as flexor digitorum profundus and flexor carpi radialis leading to the highest $\alpha_{E}$ proportion. Force signals are used for real-time grip strength adjustment resulting in the second highest $\alpha_{F}$ while joint angles only need to meet basic movement constraints leading to the lowest $\alpha_{A}$. For precision-coordinated movements such as Pinch the weights of sEMG and joint angle signals are balanced with $\alpha_{E}=0.41$ $\alpha_{A}=0.31$ and $\alpha_{F}=0.28$ the lowest. This matches the biomechanical requirements of Pinch which demands sEMG signals to transmit fine movement intent and precise coordination of metacarpophalangeal and interphalangeal joints supporting the balanced $\alpha_{E}$ and $\alpha_{A}$. Force signals only need to maintain low-intensity stable output resulting in the lowest $\alpha_{F}$ to avoid damaging objects or tissues. For joint-dominated movements such as Wrist Supination and Finger Extension the joint angle signal weight is the highest. Wrist Supination has $\alpha_{A}=0.47$ the highest and Finger Extension has $\alpha_{A}=0.43$ the second highest. Both movements have the lowest $\alpha_{F}$ values 0.15 and 0.12 respectively. This is consistent with the anatomical characteristics where Wrist Supination relies on coordinated rotation of the radiocarpal and midcarpal joints and Finger Extension depends on the coordinated movement of extensor digitorum and joint ligaments. Such movements are primarily constrained by the normativity of joint movement trajectories rather than force output intensity leading the module to prioritize joint angle signals $\alpha_{A}$ to ensure movements comply with anatomical limits. In summary the attention weight distribution of the C-CMA module is deeply tied to the biomechanical nature of hand movements. Force-dominated movements prioritize sEMG and force signals precision-coordinated movements balance sEMG and joint angle signals and joint-dominated movements focus on joint angle signals. This pattern not only validates the rationality of the C-CMA module design but also provides a clear biomechanical basis for parameter adjustment in clinical rehabilitation scenarios.

**Fig 6:**
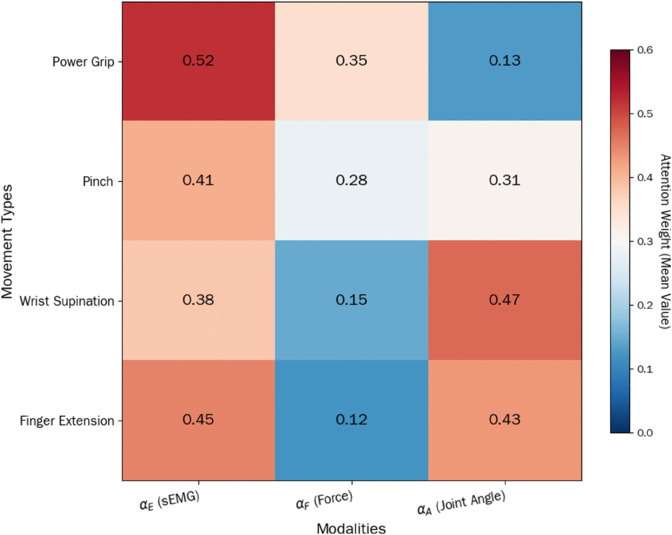
The figure shows two confusion matrices, representing the model’s action classification results on the Ninapro DB5 dataset (left) and the MUSED-I dataset (right).

As can be seen from the Fig 6, in the confusion matrix of the Ninapro DB5 dataset, the classification performance of various actions (such as fingertip pinch, cylindrical grip, wrist flexion, etc.) is outstanding. The diagonal elements dominate, indicating a high proportion of correctly classified samples. The accuracy of the “relaxing hand” action reaches 99.2%, and the fingertip pinch also achieves 95.8%. This demonstrates that the model has strong differentiation capability for healthy subjects’ actions and, with the help of multimodal data fusion, is able to precisely capture the feature differences between various actions. The confusion matrix of the MUSED-I dataset, which corresponds to unimodal data from stroke patients. Although the classification accuracy of various actions (e.g., wrist flexion, wrist extension, hand closure) is slightly lower compared to healthy data, it still maintains a high level. The accuracy of the wrist extension action is 92.9%, hand closure is 93.5%, and the “rest” action recognition accuracy reaches 97.3%, which allows for clear differentiation between active actions and static states. Overall, the model demonstrates good action classification performance on both datasets. Even when dealing with noisy unimodal EMG signals from stroke patients, it can still recognize different actions with reasonable accuracy. This performance provides reliable technical support for rehabilitation robots in both assisting daily movements of healthy individuals and controlling rehabilitation training actions in pathological scenarios. It further validates the model’s generalization ability and practicality across different data modalities and application scenarios.

**Fig 7:**
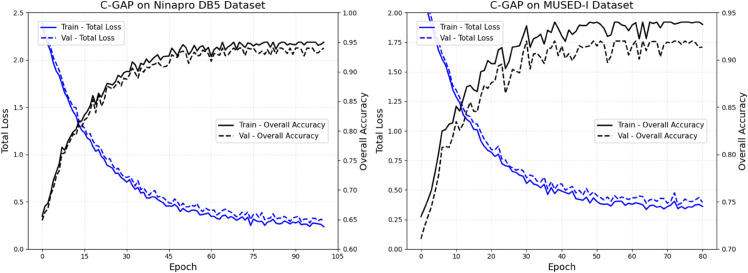
The C-GAP model’s total loss and overall accuracy as a function of training epochs (Epochs) on the Ninapro DB5 and MUSED-I datasets.

From the Fig 7, it is clear that on the Ninapro DB5 dataset, as the number of training epochs increases, both the training total loss and validation total loss steadily decrease and eventually stabilize. This trend indicates that the model’s fitting degree on the healthy subject multimodal dataset is continuously improving. At the same time, the overall training accuracy and overall validation accuracy gradually increase. The training accuracy ultimately approaches 0.95, while the validation accuracy also remains at a high level, demonstrating that the model is stable during training on the Ninapro DB5 dataset and possesses good generalization ability. On the MUSED-I dataset, the total loss and validation loss also show a decreasing trend. After a rapid rise, the overall training accuracy stabilizes around 0.95, with validation accuracy significantly improving, though slightly lower than the training accuracy, it still remains at a high level. This performance shows that the model can effectively learn on unimodal pathological data from stroke patients, demonstrating good adaptability. It can provide reliable model support for action control in rehabilitation robots under pathological scenarios.

Table 5 presents the performance differences of the complete C-GAP model and the models with key modules removed or adjusted on the datasets. The complete C-GAP model performs optimally, demonstrating its adaptability in both multimodal and pathological unimodal scenarios. When the Contrastive Cross-Modal Attention (C-CMA) module is removed, the performance on the Ninapro DB5 dataset drops significantly: the action classification accuracy decreases to 0.85 ± 0.05, the force prediction error increases to 1.92 ± 0.26 N with a 120.7% increase, the angle tracking error rises to 2.98 ± 0.42^°^ with a 121.5% increase. On MUSed-I dataset the action classification accuracy and stroke patient classification accuracy drop to 0.84 ± 0.06 and 0.80 ± 0.07 respectively. This indicates that the C-CMA module is crucial for handling multimodal data in C-GAP. Its absence results in the inability to precisely correlate EMG, force, and angle features, directly impairing the multimodal collaborative modeling ability. When the Stacked Gated Recurrent Units (Stacked GRU) module is removed, the performance on both datasets is impacted: the action classification accuracy on Ninapro DB5 drops to 0.88 ± 0.04, the force prediction error increases to 1.38 ± 0.17 N with a 58.6% increase, the angle tracking error rises to 2.15 ± 0.31^°^ with a 59.3% increase. On MUSed-I the action classification accuracy and stroke patient classification accuracy drop to 0.86 ± 0.05 and 0.82 ± 0.06 respectively. Further ablation on Stacked GRU architecture shows that replacing Stacked GRU with single-layer GRU leads to moderate performance degradation: on Ninapro DB5 the action classification accuracy is 0.89 ± 0.04, the force prediction error is 1.25 ± 0.16 N with a 43.7% increase compared to complete model, the angle tracking error is 1.98 ± 0.28^°^ with a 46.7% increase. On MUSed-I the action classification accuracy and stroke patient classification accuracy are 0.87 ± 0.05 and 0.83 ± 0.06 respectively. Removing residual connections from Stacked GRU results in slight performance reduction: on Ninapro DB5 the action classification accuracy is 0.92 ± 0.03, the force prediction error is 1.02 ± 0.14 N with a 17.2% increase, the angle tracking error is 1.65 ± 0.23^°^ with a 22.2% increase. On MUSed-I the action classification accuracy and stroke patient classification accuracy are 0.90 ± 0.04 and 0.86 ± 0.05 respectively. This shows that Stacked GRU is crucial for capturing the temporal dynamics of actions. Stacked depth enhances the modeling of hierarchical temporal dependencies in motion sequences while residual connections alleviate gradient vanishing in deep recurrent networks, both contributing to the superior performance of the complete model. Without stacked structure the model fails to effectively learn the continuous change features of action sequences, leading to a decline in both classification and control accuracy. When the Adaptive Proportional-Integral-Derivative (Adaptive PID) module is removed, the control performance of the model is significantly weakened: the force prediction error on Ninapro DB5 increases to 1.56 ± 0.20 N with a 79.3% increase, the angle tracking error rises to 1.89 ± 0.28^°^ with a 40.0% increase. Although the action classification accuracy slightly decreases to 0.91 ± 0.03 it still remains high. On MUSed-I the action classification accuracy and stroke patient classification accuracy drop to 0.86 ± 0.05 and 0.82 ± 0.06 respectively. This shows that the Adaptive PID module primarily optimizes force control accuracy and trajectory smoothness. Its absence has a smaller impact on the classification logic but directly decreases the stability of rehabilitation robot control, which is highly relevant to the clinical training requirement of precise force control to avoid secondary injuries. The three key modules of the C-GAP model C-CMA, Stacked GRU, Adaptive PID work synergistically: C-CMA ensures multimodal feature alignment, Stacked GRU enhances temporal action modeling with stacked depth and residual connections, and Adaptive PID optimizes control output accuracy. Together, they support the model’s high performance in both healthy human multimodal interaction and stroke patient pathological rehabilitation scenarios, validating the rationality and necessity of the overall C-GAP architecture design.

**Table 5:** Ablation study: Performance comparison of different model configurations on Ninapro DB5 and MUSed-I datasets.

Model Configuration	Ninapro DB5 Dataset			MUSed-I Dataset	
	Action Classification Accuracy (ACC)	Force Prediction Error (MAE_F, N)	Angle Tracking Error (MAE_θ, ^∘^)	Action Classification Accuracy (ACC)	Stroke Patient Classification Accuracy
Complete Model (C-GAP)	0.94 ± 0.03	0.87 ± 0.12	1.35 ± 0.21	0.92 ± 0.04	0.89 ± 0.05
w/o C-CMA	0.85 ± 0.05	1.92 ± 0.26	2.98 ± 0.42	0.84 ± 0.06	0.80 ± 0.07
w/o Stacked GRU	0.88 ± 0.04	1.38 ± 0.17	2.15 ± 0.31	0.86 ± 0.05	0.82 ± 0.06
w/ Single-Layer GRU	0.89 ± 0.04	1.25 ± 0.16	1.98 ± 0.28	0.87 ± 0.05	0.83 ± 0.06
(w/o Stacked Depth)					
w/ Stacked GRU	0.92 ± 0.03	1.02 ± 0.14	1.65 ± 0.23	0.90 ± 0.04	0.86 ± 0.05
(w/o Residual)					
w/o Adaptive PID	0.91 ± 0.03	1.56 ± 0.20	1.89 ± 0.28	0.86 ± 0.05	0.82 ± 0.06

## Discussion

This study proposed a novel approach integrating contrastive cross-modal attention and stacked GRU for hand function rehabilitation robot control. The results obtained from this method have opened up several critical aspects for discussion. The model’s performance demonstrates the feasibility of leveraging multi-modal data fusion and temporal modeling techniques in the context of rehabilitation robotics, indicating that the combination of these methods can enhance the accuracy and responsiveness of robot control, thereby providing more effective rehabilitation assistance.

However, the outcomes also expose certain limitations. Despite the promising results, the model’s generalization ability across different patient populations with diverse impairment degrees still needs improvement. Individual differences in physiological characteristics, the severity of the condition, and unique movement patterns can influence the model’s effectiveness. Furthermore, the current parameter settings of the adaptive PID control module have not been verified against formal clinical rehabilitation protocols and patient-specific clinical data. While these values are set to align with the natural physiological motion rules of human hands, their direct applicability to clinical rehabilitation scenarios for stroke patients still requires further evaluation and optimization with clinical empirical evidence. Additionally, in practical application scenarios, the system may face challenges from environmental noise and sensor instability, which can potentially disrupt the accuracy of the control signals.

In the future, there are multiple directions worthy of exploration. Research efforts could be directed towards developing more personalized calibration strategies to adapt the model to individual patients. Exploring more advanced noise reduction and sensor fusion techniques may also enhance the system’s robustness in real-world environments. Importantly, we plan to collaborate with clinical rehabilitation professionals to conduct targeted validation and optimization of the control strategy. We aim to refine the control parameters with reference to clinical rehabilitation experience and real patient training feedback, and further verify the safety and efficacy of the proposed method in practical rehabilitation training sessions. At the same time, we will further improve the generalization ability of the model by expanding the sample size covering patients with different impairment levels and integrating individualized motion feature modeling for hemiplegic upper limbs. Overall, although significant progress has been made, continuous research and optimization are necessary to fully realize the potential of this approach and bring more substantial benefits to hand function rehabilitation practice.
